# Predicting Glycerophosphoinositol Identities in Lipidomic Datasets Using VaLID (Visualization and Phospholipid Identification)—An Online Bioinformatic Search Engine

**DOI:** 10.1155/2014/818670

**Published:** 2014-02-20

**Authors:** Graeme S. V. McDowell, Alexandre P. Blanchard, Graeme P. Taylor, Daniel Figeys, Stephen Fai, Steffany A. L. Bennett

**Affiliations:** ^1^Neural Regeneration Laboratory, Department of Biochemistry, Microbiology, and Immunology, University of Ottawa, ON, Canada K1H 8M5; ^2^Ottawa Institute of Systems Biology, Department of Biochemistry, Microbiology, and Immunology, University of Ottawa, ON, Canada K1H 8M5; ^3^CIHR Training Program in Neurodegenerative Lipidomics, Department of Biochemistry, Microbiology, and Immunology, University of Ottawa, ON, Canada K1H 8M5; ^4^Carleton Immersive Media Studio, Azrieli School of Architecture and Urbanism, Carleton University, ON, Canada K1S 5B6

## Abstract

The capacity to predict and visualize all theoretically possible glycerophospholipid molecular identities present in lipidomic datasets is currently limited. To address this issue, we expanded the search-engine and compositional databases of the online Visualization and Phospholipid Identification (VaLID) bioinformatic tool to include the glycerophosphoinositol superfamily. VaLID v1.0.0 originally allowed exact and average mass libraries of 736,584 individual species from eight phospholipid classes: glycerophosphates, glyceropyrophosphates, glycerophosphocholines, glycerophosphoethanolamines, glycerophosphoglycerols, glycerophosphoglycerophosphates, glycerophosphoserines, and cytidine 5′-diphosphate 1,2-diacyl-sn-glycerols to be searched for any mass to charge value (with adjustable tolerance levels) under a variety of mass spectrometry conditions. Here, we describe an update that now includes all possible glycerophosphoinositols, glycerophosphoinositol monophosphates, glycerophosphoinositol bisphosphates, and glycerophosphoinositol trisphosphates. This update expands the total number of lipid species represented in the VaLID v2.0.0 database to 1,473,168 phospholipids. Each phospholipid can be generated in skeletal representation. A subset of species curated by the Canadian Institutes of Health Research Training Program in Neurodegenerative Lipidomics (CTPNL) team is provided as an array of high-resolution structures. VaLID is freely available and responds to all users through the CTPNL resources web site.

## 1. Introduction

The emerging field of lipidomics seeks to answer two seemingly simple questions: How many lipid species are there? What effect does lipid diversity have on cellular function? To address these questions, lipidomics requires a comprehensive assessment of cellular, regional, and systemic lipid homeostasis. This assessment expands beyond lipid profiling to include the transcriptomes and proteomes of lipid metabolic enzymes and transporters, as well as that of the protein targets that affect downstream lipid signalling [1]. Lipidomic analyses also encompass an unbiased mechanistic assessment of lipid function ranging from the physicochemical basis of lipid behaviour to lipid-protein and lipid-lipid interactions triggered by intrinsic and extrinsic stimuli [1]. The first step, however, lies in identifying the molecular identities of the lipid constituents in different membrane compartments.

Recent technological advances in electrospray ionization (ESI) and matrix-assisted laser desorption ionization (MALDI) mass spectrometry (MS), coupled to high performance liquid chromatography (LC), allow lipid diversity and membrane composition to be quantified at the molecular level [4–7]. Thousands of unique lipid species across the six major lipid structural categories in mammalian cells (fatty acyls, glycerolipids, glycerophospholipids, sphingolipids, sterol lipids, and prenol lipids) and two lipid categories synthesized by other organisms (saccharolipids and polyketides) can now be identified using LC-ESI-MS and, in some cases, MALDI-MS imaging [1, 4, 8]. Yet, with these successes come new challenges. Turning raw MS spectral data into annotated lipidomic datasets is a time-consuming, labour-intensive, and highly inefficient process. Predicting identities of “new” species, not previously curated, is exceedingly difficult. Lipidomic investigations lack essential bioinformatic tools capable of enabling automated data processing and exploiting the rich compositional data present in MS lipid spectra.

**Figure 1:**
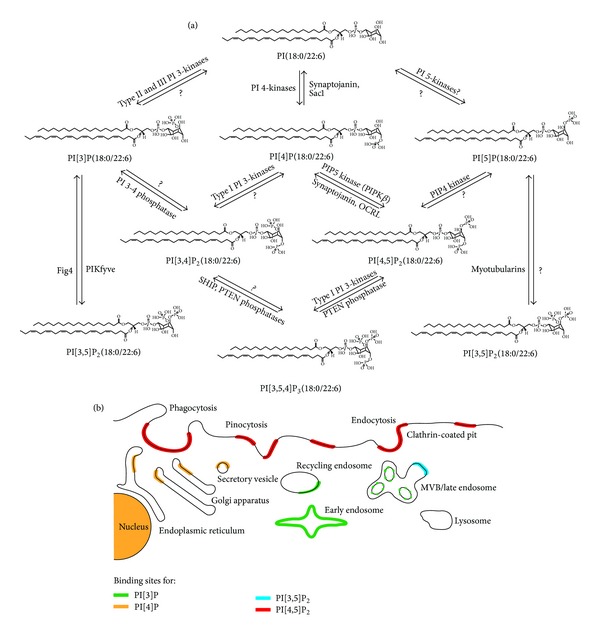
Glycerophosphoinositol (PI) metabolism to PI phosphates (PIP_x_). (a) Metabolism of membrane PIs to bioactive PIP_x_ second messengers. The molecular identity of each species, defined by carbon chain length and linkage to the glycerophospholipid backbone, is predicted to affect signalling specificity in addition to known effects of PI headgroup phosphorylation. (b) Phosphorylation of PIP_x_ species regulates the localization of different PI-binding proteins and targets them to specific organelles (i.e., lipid-protein interaction). Phosphorylation status and carbon chain length dictate localization and likely restrict functions. Together, structural PIs and their PIP_x_ second messengers regulate vesicular fusion, exocytosis, and endocytosis as reviewed in (and adapted from) [2, 3].

**Figure 2:**
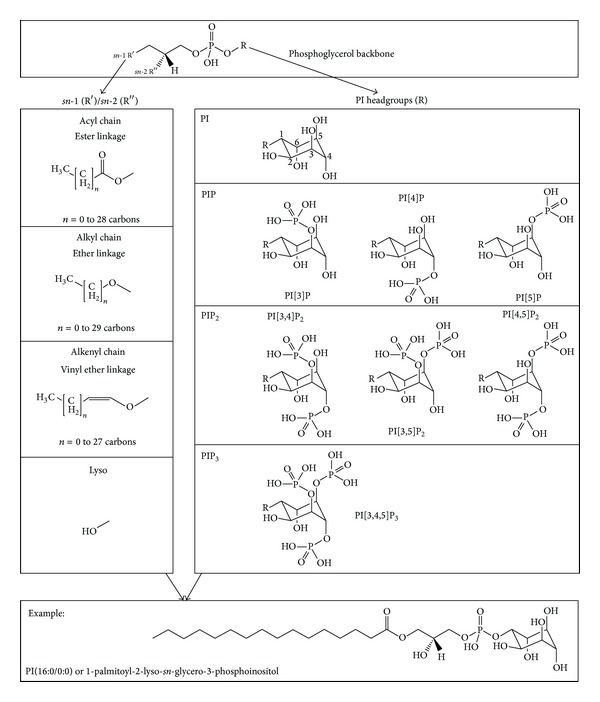
Component and composite structural PI and PIP_x_ features used to calculate masses. Exact and average masses for all theoretically possible PI and PIP_x_ species were calculated from the masses of every component possibility: (top panel) the phosphoglycerol backbone, (left panel) *sn-*1 and *sn-*2 hydroxyl residues (lyso-lipids) and *sn-1* and *sn-2 *fatty chains ranging from 0 to 30 carbons with up to six unsaturations, considering ester, ether, or vinyl ether linkages to the phosphoglycerol backbone, and (right panel) PI polar headgroups and all biologically relevant phosphorylation possibilities. The bottom panel provides one composite PI example.

The critical first step is to unambiguously assign molecular identities from the MS structural information present in large lipidomic datasets [9]. Where genomics and proteomics capitalize on sequence-based signatures, lipids lack such easily definable molecular fingerprints. Identities must be reconstructed by analysis of (a) lipid mass to charge (*m/z*) ratios following “soft” ionization ESI and MALDI techniques and (b) defining fragmentation patterns obtained after collision-induced dissociation in various MS modes [7]. Once these molecular identities are predicted, further information about stereospecificity of critical species can then be assessed (e.g., by tandem MS, analysis of *lyso*-form fragment ions, and product ion spectral evaluation) [10–13]. For example, membrane phospholipids are derivatives of *sn*-glycero-3-phosphate with (a) an acyl, an alkyl (ether-linked plasmanyl), or an alkenyl (alkyl-1′-enyl, vinyl ether-linked plasmenyl) carbon chain at the *sn*-1 position; (b) a long-chain fatty acid that is usually esterified to the *sn-*2 position; and (c) a polar headgroup composed of a nitrogenous base, a glycerol, or an inositol unit modifying the phosphate group at the *sn*-3 position. The polar head group defines membership in one of 20 different phospholipid classes (e.g., glycerophosphoserines (PS), glycerophosphoethanolamines (PE), glycerophosphocholines (PC), glycerophosphoinositols (PI), etc.) [14]. Molecular species are further distinguished by individual combinations of carbon residues (chain length and degree of unsaturation) and the nature of each *sn*-1 or *sn-*2 chemical linkage (acyl, alkyl, or alkenyl) to the glycerol backbone. PI(18:0/22:6), for example, defines a lipid with a phosphoinositol polar head group (PI), a fully saturated 18 carbon chain (referred to as  :0) ester-linked at the *sn*-1 position, and a 22 carbon chain which is characterized by six unsaturations (indicated by  :6) ester-linked at the *sn*-2 position (Figure 1). Immediate PI metabolites (PIP_x_) are then produced by carbon-specific phosphorylation of the PI headgroup with unique fatty acyl, alkyl, and/or alkenyl *sn*-1 and *sn*-2 chains (Figures 1 and 2). The tight regulation of PI metabolism and its critical impact on cellular function clearly underlines the importance of these compositional changes (Figure 1). Yet, to date, biological significance of the astonishing number of potentially unique PIs and PIP_x_s is unknown. This is primarily due to the challenges associated with unambiguous compositional identification of PIs and PIP_x_s in biological membranes [1, 15–19].

Key advances in lipidomic bioinformatics have been led by the LIPID MAPS consortium both in the development of online spectral databases and the reorganization of lipid class ontologies [14, 20]. These toolsets and classification systems have recently been complemented by the *in silico* generation of a searchable library of all theoretically possible MS/MS lipid spectra in different ionization modes (LipidBlast) [21]. Such fundamental toolkits are supported by a growing compendium of targeted spectral tools, reviewed in [6, 7, 20, 22]. Few existing bioinformatic resources, however, provide necessary information on all potential acyl chain inversions (e.g., *sn*-1 versus *sn*-2), critical phospholipid linkages that define lipid function, or theoretically possible double bond positions for every possible species. To address this need, we have developed Visualization and Phospholipid Identification (VaLID)—a web-based application linking a user-friendly online search engine, structural composition database, and multiple visualization features—that is capable of providing users with all theoretically possible phospholipids calculated from any *m/z* under a variety of MS conditions. VaLID version 1.0.0 was initially restricted to 736,584 unique PS, PE, PC, glycerophosphate (PA), glyceropyrophosphate (PPA), glycerophosphoglycerol (PG), glycerophosphoglycerophosphate (PGP), and cytidine 5′-diphosphate 1,2-diacyl-*sn*-glycerol (CDP-DG) identities (Table 1) [22]. At first release, we did not include the PI family or their bioactive PIP_x_ metabolites given the significant challenges associated with automating the visualization of all theoretically possible combinations of *sn*-1 and *sn-*2 carbon chain lengths, linkages, and variations in phosphorylation of the phosphoinositol head group. Here, we address this deficit through the development of VaLID version 2.0.0, now coded with an exhaustive PI and PIP_x_ database, capable of computing and visualizing a total of 1,473,168 theoretically possible phospholipids predicted from any user-inputted *m/z* value and MS condition. VaLID version 2.0.0 is freely available for commercial and noncommercial use at http://neurolipidomics.ca and http://neurolipidomics.com/resources.html.

## 2. Materials and Methods

### 2.1. Programming Language and Packages

VaLID version 2.0.0. was developed using Oracle's Java programming language version 6 and external Java libraries from JExcelApi and structures are displayed within the program by ChemAxon's Marvin View 5.5.1.0. software. The code was written using the IDE Eclipse Kepler, and packaged using the Fat Jar Eclipse version 0.0.31 plugin. VaLID is a web-based Java applet, and thus it requires that Java be both installed and enabled on a user's web browser. The most recent Java security update is recommended, and can be downloaded from http://www.oracle.com/technetwork/java/index.html.

### 2.2. The PI and PIP_x_ Compositional Database

Briefly, the underlying database contains masses of all theoretically possible PI and PIP_x_ species calculated from both exact and average atomic masses [23]. Component structural masses were first established for: (a) the glycerol backbone, (b) PI polar headgroups with all phosphorylation possibilities, (c) *sn-*1 and *sn-*2 hydroxyl residues (lyso-lipids), (d) *sn-*1 and *sn-*2 fatty chains ranging from 0 to 30 carbons with up to six unsaturations, considering (e) ester, ether, or vinyl ether linkages to the phosphoglyceride backbone (Figure 2). Composite masses were then calculated for every theoretically possible combination. Thus, the underlying database includes all PIs, as well as every acyl, alkyl, and alkenyl variant, for every carbon chain and double bond position, of all mono- (PIP), bis- (PIP_2_), and tris- (PIP_3_) phosphorylated PI headgroups modified on the hydroxyl group of carbons 3, 4, and/or 5.

### 2.3. PI and PIP_x_ Structural Visualizations

We have updated the automated representation drawing feature of VaLID to be able to draw all theoretically possible PI and PIP_x_ molecular identities. Structures have been restricted to display only cis double bonds separated by a minimum of two carbons. To achieve this goal, the basic structure of the PI backbone was created manually and the atom placement corrected mathematically to match known structures. Slight adjustments to atom placement were further made to improve visibility. The locations of each atom in the headgroup were then established on a Cartesian plane and coded into the software. The automated drawing feature update was integrated into the database and search functions, allowing all PI and PIP_x_ to be visualized on demand. Chemical structures are displayed using ChemAxon's MarvinView software (Marvin 5.5.1.0, 2011, http://www.chemaxon.com).

## 3. Results and Discussion

PI and PIP_x_ are derivatives of *sn*-glycero-3-phosphate with (a) an acyl, an alkyl (ether-linked plasmanyl), or an alkenyl (alkyl-1′-enyl, vinyl ether-linked plasmenyl) carbon chain; (b) a fatty acid commonly esterified but also with possible alkyl or alkenyl linkages to the *sn-*2 position; and (c) a polar headgroup composed of an inositol unit modifying the phosphate group at the *sn*-3 position. Individual species are distinguished by their particular combination of carbon chains (chain length and degree of unsaturation) and by the nature of their *sn*-1 or *sn-*2 chemical linkages (acyl, alkyl, or alkenyl). PI[3,4, 5]P_3_(*O*-16:0/20:4), for example, defines a lipid species with a phosphoinositol polar head group (PI) phosphorylated at the 3rd, 4th, and 5th carbon positions, an ether linkage at the *sn-*1 position (*O*-), 16 carbons at the *sn*-1, and 20 carbons at the *sn*-2 positions, of which the *sn*-1 chain is fully saturated. The number of possible structural and biochemical combinations results in colossal structural diversity; however, PIP_x_ lipids account for less than 15 percent of the total phospholipid composition in eukaryote cells [24]. The molecular identities of these critical species have yet to be determined in different lipidomes despite emerging evidence that differences in carbon chain length, linkage, and phosphorylation status fundamentally alter biological activity [1, 15–19] (Figure 1).

**Figure 3:**
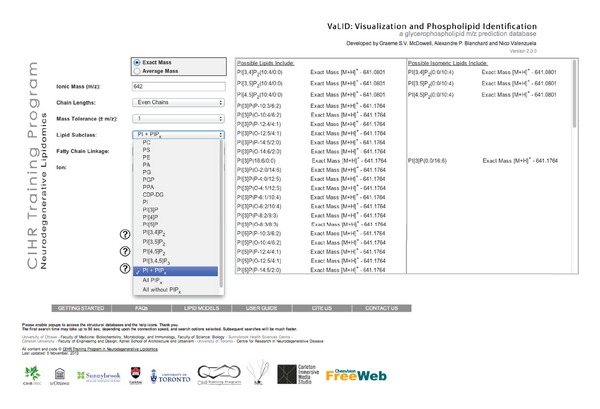
VaLID 2.0.0 interface. The new PI and PIP_x_ search options are shown for the VaLID interface's drop down menu. Each member of the PI family can be searched individually, as well as in various combinations. PIP_x_ refers to phosphoinositol mono-, bis-, or tris-phosphate, and can be searched with, or without, PIs.

Here, we enhanced VaLID's capacity to (a) predict identities of glycerophosphoinositol species present in MS spectra from *m/z* under user-defined MS conditions and (b) automatically visualize every theoretically possible PI molecular species at given *m/z*. The updated VaLID interface, showing all of the available search terms, is presented in Figure 3. Since its inception, VaLID was designed to be a comprehensive glycerophospholipid database linking a convenient search engine with visualization features for identification and dissemination of large-scale lipidomic datasets. The intent of this tool was to aid in lipid discovery obtained through multiple MS methodologies and significantly reduce the time required to validate critical phospholipid identities present in target lipidomes. The program initially contained eight phospholipid subclasses, excluding the PI subfamily. In VaLID version 2.0.0, this capacity is now expanded to all theoretically possible PI and PIP_x_ glycerophospholipids and comprises a total of 1,473,168 unique structures. These additions are meant to provide lipidomic researchers with the additional tools necessary to mine their lipidomes for PI and PIP_x_ species with specific *m/z* under their particular MS experimental conditions including the ion mode and the lipid subclass. Due to the complexity of the PI superfamily, and to accelerate searching, users can restrict searches to subclasses (PI, PIP_x_) or sub-subclasses (PI, PI[3]P, PI[4]P, PI[5]P, PI[3,4]P_2_, PI[3,5]P_2_, PI[4,5]P_2_, PI[3,4, 5]P_3_). For example, if the option PI[3,4]P_2_ is chosen, all molecular species with an inositol backbone phosphorylated only at the 3rd and 4th carbon positions will be provided and VaLID will not return any related PI[3,5]P_2_ or PI[4,5]P_2_ species. The PI +PIP_x_ option restricts searches to the entire PI superfamily excluding other phospholipid families. The “All without the PIP_x_” option returns all of the phospholipids in the database including PI structural precursors with the exception of PIP_x_ metabolites. Finally, the “All” option returns results from every headgroup. When more than one headgroup is being searched, the program will let the user know how many headgroups have been loaded, and how many are remaining to be loaded.

**Figure 4:**
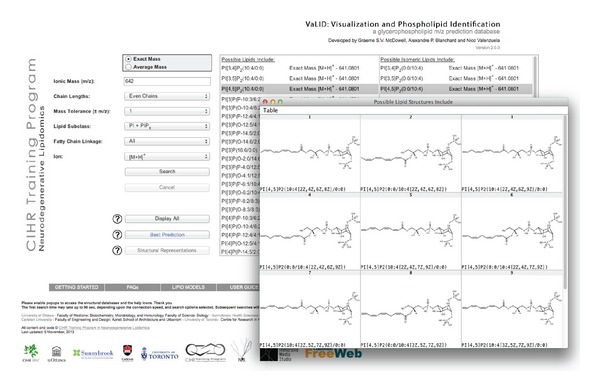
Automated drawing feature of VaLID 2.0.0. An example of a search button, returning all possible PI and PIP_x_ lipids with *m/z* of 642 (exact mass with a user-defined tolerance of 1 amu), restricted to displaying even carbon chains only, and selecting [M+H]^+^ ion mode in MS (back panel). The user then selected PI[4,5]P_2_(10:4/0:0) and its *sn-*1/*sn*-2 chain inversion species and pressed “Display All” button. The window labelled “Possible Lipid Structures Include” displays a table containing the possible structures for this lipid, with the restrictions as laid out in the user manual (inset). These drawings can be easily exported for use in publication figures as described in the user manual.

With respect to the visualization features for PIP or PIP_2_, the program will draw the phosphate groups on the inositol ring in the locations that the user specified from the dropdown menu for lipid species selected. As with the other subclasses, choosing the “Display All” button will draw all the theoretically possible structures associated with the selected lipid name. Potential variants in degrees of unsaturation are drawn sequentially in every location along the fatty acid chain, separated by at least two carbons, and in cis configuration. An example of this can be seen in Figure 4. If the selected lipid meets criteria for the “Best Prediction,” selecting this option will return only the lipids in VaLID's “Predicted to be Common” database. These species are categorized based on the relative abundance of prevalent fatty acid chains in mammalian cells [25].

## 4. Conclusions

VaLID is, to our knowledge, the first search engine that has an exhaustive *m/z* and visualization database of all the theoretically possible glycerophospholipids updated here from eight to twelve of the twenty phospholipid subclasses defined by the LIPID MAPS Consortium [26]. The purpose of this update is to facilitate prediction and visualization of the identities of all unknown species, now including all PIs and their metabolites, with given *m/z* and MS condition that may be present in users' lipidomes.

## Figures and Tables

**Table 1:** Total number of species from each subclass that is included in VaLID.

Phospholipid subclass	LIPIDMAPS classification	Abbreviation	Number of species*
Glycerophosphates	GP10	PA	92073
Glyceropyrophosphates	GP11	PPA	92073
Glycerophosphocholines	GP01	PC	92073
Glycerophosphoethanolamines	GP02	PE	92073
Glycerophosphoglycerols	GP04	PG	92073
Glycerophosphoglycerophosphates	GP05	PGP	92073
Glycerophosphoinositols	GP06-09	PI, PIP_x_	736584
Glycerophosphoserines	GP03	PS	92073
Cytidine 5′-diphosphate glycerols	GP13	CDP-DG	92073

		Total	1473168

*The calculated number of species does not include lipids formed by changing the position of the double bond beyond those represented in VaLID's structural models. Each lipid *m/z* has been calculated for exact and average masses and can be searched using even and odd carbon chains with mass tolerance ranging from ±0.0001 to ±2 and MS ion modes [M + H]^+^, [M + K]^+^, [M + Li]^+^, [M + Na]^+^, [M – H]^−^, or [M (Neutral)].
